# Antibiotic Resistance Profiles of Invasive Bacterial Isolates from Hospitalized Pediatric Patients in Serbia: A Multicenter Surveillance Study, 2020–2024

**DOI:** 10.3390/antibiotics15070694

**Published:** 2026-07-16

**Authors:** Deana Medic, Dusan Mihajlo Spasic, Marija Kukuric, Milica Bajcetic

**Affiliations:** 1 Department of Microbiology with Parasitology and Immunology, Faculty of Medicine, University of Novi Sad, 21000 Novi Sad, Serbia; deana.medic@mf.uns.ac.rs; 2 Institute of Public Health of Vojvodina, Center for Microbiology and Virology, 21000 Novi Sad, Serbia; 3 Department of Pharmacology, Clinical Pharmacology and Toxicology, Faculty of Medicine, University of Belgrade, 11000 Belgrade, Serbia; dusan.spasic.dr@med.bg.ac.rs; 4 Department of Emergency Radiology, University Clinical Center of Serbia, 11000 Belgrade, Serbia; marija.kukuric@kcs.ac.rs

**Keywords:** antimicrobial resistance, pediatrics, bloodstream infection, cerebrospinal fluid, *Klebsiella pneumoniae*, ESBL, carbapenem resistance, Serbia, CAESAR

## Abstract

**Background**: Antimicrobial resistance (AMR) threatens empirical therapy for invasive pediatric infections, but contemporary data from Serbia and the Western Balkans remain limited for blood and cerebrospinal fluid isolates. **Methods**: We performed a retrospective CAESAR-linked multicenter microbiological surveillance analysis of first eligible invasive bacterial isolates from hospitalized children in Serbia from 1 January 2020 to 31 December 2024. Organism distribution, ESBL production, carbapenemase marker results, and selected resistance outcomes were analyzed descriptively and with temporal trend and multivariable models. **Results**: Among 907 isolates, 879 (96.9%) were recovered from blood and 28 (3.1%) from cerebrospinal fluid; neonates and infants accounted for 57.9% of isolates. *K. pneumoniae* was the predominant organism (318/907, 35.1%), followed by *E. coli* (172/907, 19.0%), *S. aureus* (137/907, 15.1%), and *Acinetobacter* spp. (96/907, 10.6%). Among *K. pneumoniae* and *E. coli* isolates with available ESBL results, ESBL production was frequent, whereas carbapenemase testing was restricted to a selected subset and the carbapenemase type was not recorded in the national surveillance extract. In *K. pneumoniae*, meropenem resistance increased from 19.7% in 2020 to 48.6% in 2024, and later study years remained independently associated with meropenem resistance after adjustment. **Conclusions**: This microbiological surveillance study identifies *K. pneumoniae* predominance, neonatal concentration, frequent ESBL production, and a rising carbapenem-resistance signal as the principal findings among invasive pediatric isolates in Serbia. Resistance estimates, particularly carbapenemase production, should be interpreted as surveillance indicators rather than clinical prevalence estimates. These data support strengthened pediatric invasive-isolate surveillance, center- and age-specific antibiograms, molecular characterization, and coordinated antimicrobial stewardship and infection prevention and control efforts within the wider European AMR context.

## 1. Introduction

Antimicrobial resistance (AMR) is one of the defining threats to contemporary infectious-disease practice because it progressively reduces the reliability of empirical and targeted antibacterial therapy. Global estimates for 2019 attributed 1.27 million deaths directly to bacterial AMR and associated 4.95 million deaths with drug-resistant bacterial infections, while updated analyses through 2021 confirmed that AMR remains a major cause of mortality with a projected long-term burden extending to 2050 [1,2]. In Europe, population-level modeling has similarly shown a substantial burden of infections caused by antibiotic-resistant bacteria, with particularly high impact at the extremes of age [3]. These data have made AMR surveillance a central component of public-health strategy rather than a purely laboratory-based exercise.

Children represent a clinically distinct AMR population given their age-dependent immune vulnerability, frequent infectious exposures, and high probability of receiving empirical antimicrobial therapy before definitive microbiological confirmation. The global burden of pediatric and neonatal sepsis remains substantial, with estimates of millions of neonatal and pediatric sepsis cases annually, and mortality is particularly pronounced in neonates and settings with limited access to timely diagnostics and effective antimicrobials [4]. At the same time, respiratory infections and otitis media are among the most frequent acute illnesses in childhood, and their epidemiologic frequency contributes to high antibiotic exposure even though the majority of episodes are viral or self-limiting [5]. Broader analyses of human antibiotic consumption further emphasize that antimicrobial use has increased globally, thereby creating selective pressure that is especially relevant in settings where broad-spectrum agents are commonly used [6].

The clinical consequences of resistance are greatest when infection is invasive. Bloodstream and central nervous system infections require immediate treatment decisions, and the initial regimen may determine whether early therapy is effective. Therefore, AMR surveillance based on invasive isolates from blood and cerebrospinal fluid is particularly valuable for empirical-therapy policy. The European Antimicrobial Resistance Surveillance Network (EARS-Net) and the Central Asian and European Surveillance of Antimicrobial Resistance (CAESAR) network both prioritize routine antimicrobial susceptibility data from invasive isolates, enabling comparisons across countries and over time [7,8,9].

Available pediatric surveillance studies show that resistance patterns differ markedly by pathogen, age group, region, and clinical setting. In European pediatric Gram-negative isolates, the ATLAS (Antimicrobial Testing Leadership and Surveillance) surveillance study reported increasing extended-spectrum beta-lactamase (ESBL) and carbapenem-resistance phenotypes in *Klebsiella pneumoniae* (*K. pneumoniae*) and increasing carbapenem resistance in *Acinetobacter baumannii* (*A. baumannii*) and *Pseudomonas aeruginosa* (*P. aeruginosa*), whereas ESBL phenotypes in *Escherichia coli* (*E. coli*) decreased over time [10]. In high-complexity pediatric units in Madrid, bloodstream-isolate surveillance documented a high AMR burden, with increasing multidrug resistance and carbapenem resistance among *Enterobacterales* [11]. These findings indicate that pediatric AMR cannot be reliably inferred from adult data alone and requires age-specific and setting-specific surveillance.

In Serbia, AMR should be considered within the combined context of antibiotic consumption, antimicrobial stewardship, and hospital-based invasive-pathogen surveillance. A Serbian analysis of antibiotic consumption and invasive hospital pathogens found significant correlations between consumption of meropenem, ertapenem, ceftriaxone, and levofloxacin and resistance in invasive *K. pneumoniae* and *E. coli* isolates [12]. In pediatric primary care, a long-term public antibiotic-awareness campaign significantly reduced inappropriate antibiotic use, demonstrating that coordinated stewardship interventions can alter prescribing behavior [13]. However, improvements in outpatient prescribing do not eliminate the need for monitoring invasive hospital pathogens, especially in neonatal and intensive-care environments where colonization pressure, device exposure, empirical broad-spectrum therapy, and cross-transmission can sustain resistant Gram-negative organisms [14,15].

The Western Balkans have also been identified as an important region for carbapenem-resistant *Enterobacterales* surveillance because gaps in the European AMR map can obscure the true regional burden of carbapenem resistance [16]. This is particularly relevant for pediatric invasive infections because ESBL production, carbapenemase production, and carbapenem resistance narrow therapeutic options and may indicate the circulation of hospital-adapted strains. The WHO bacterial priority-pathogen framework reinforces the public-health importance of resistant *Enterobacterales*, *A. baumannii*, *P. aeruginosa*, and *Staphylococcus aureus* (*S. aureus*) [17].

However, several important gaps remain. First, most AMR surveillance outputs are not pediatric-specific and may not reflect pathogen distribution in neonates, infants, and high-risk pediatric wards. Second, adult invasive-isolate trends cannot be directly extrapolated to hospitalized children because exposure patterns, ward ecology, empirical regimens, and host vulnerability differ substantially. Third, in Serbia and the Western Balkans, contemporary multicenter data focused specifically on pediatric blood and cerebrospinal fluid isolates remain scarce, and available surveillance datasets are rarely linked to molecular, antimicrobial-consumption, colonization-screening, infection-control, and patient-outcome data. The present study addresses part of this gap by providing contemporary multicenter microbiological surveillance data for invasive pediatric isolates in Serbia, while explicitly recognizing the limitations of isolate-level surveillance.

## 2. Results

### 2.1. Overall Characteristics of the Isolate and Dataset

A total of 907 bacterial isolates recovered from blood or cerebrospinal fluid specimens obtained from hospitalized pediatric patients were included in the analysis. Of these, 503 (55.5%) were obtained from male patients and 404 (44.5%) from female patients. Most isolates originated from blood samples (879/907, 96.9%), whereas 28 (3.1%) were recovered from cerebrospinal fluid.

The median age was 0.33 years (IQR 0.03–1.00). Stratification by pediatric age group showed that 286 isolates (31.5%) were obtained from neonates, 239 (26.4%) from infants, 255 (28.1%) from children aged 1–5 years, 50 (5.5%) from those aged 6–11 years, and 77 (8.5%) from adolescents aged 12–17 years.

The annual number of isolate entries ranged from 154 in 2022 to 217 in 2021. By center, the largest proportions of entries were contributed by the Institute of Public Health of Vojvodina, Novi Sad (Center Novi Sad) (316/907, 34.8%) and University Children’s Hospital Tiršova (Center Tiršova) (279/907, 30.8%), followed by University Clinical Centre Kragujevac (Center Kragujevac) (83/907, 9.2%) and Institute of Public Health Niš (Center Niš) (49/907, 5.4%). Regarding hospital departments, most entries were obtained from Pediatrics/Neonatology (PED/NEO) (685/907, 75.5%), followed by Pediatric Intensive Care Unit (PEDICU) (70/907, 7.7%) and Surgery (SURG) (52/907, 5.7%). The baseline characteristics of isolate entries are summarized in Supplementary Table S1.

The analytic dataset represented routine microbiological surveillance isolate rather than clinically adjudicated infection episodes. Core variables used for the primary descriptive analyses included age, sex, specimen type, specimen date, organism, reporting center, and hospital department. ESBL results were analyzed only for *K. pneumoniae* and *E. coli* isolates with valid marker results, while carbapenemase results were interpreted only among isolates for which confirmatory testing was recorded. Missing, not tested, and not applicable results were not imputed and were excluded from tested denominators. Therefore, all resistance percentages in the manuscript should be interpreted with reference to the relevant tested denominator. Dataset completeness and testing denominators are summarized in Supplementary Table S6.

**Figure 1:**
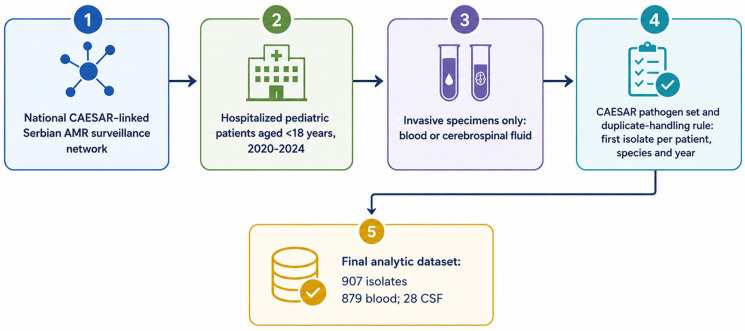
Study selection and analysis workflow. The diagram summarizes the retrospective CAESAR-linked surveillance extract, eligibility criteria, invasive specimen sources, included pathogen groups, duplicate-handling rule, and final analytic dataset. The final dataset included 907 isolate from hospitalized patients aged <18 years, including 879 blood isolates and 28 cerebrospinal fluid isolates.

The study selection process and analysis workflow are summarized in Figure 1.

### 2.2. Distribution of Bacterial Isolates

**Figure 2:**
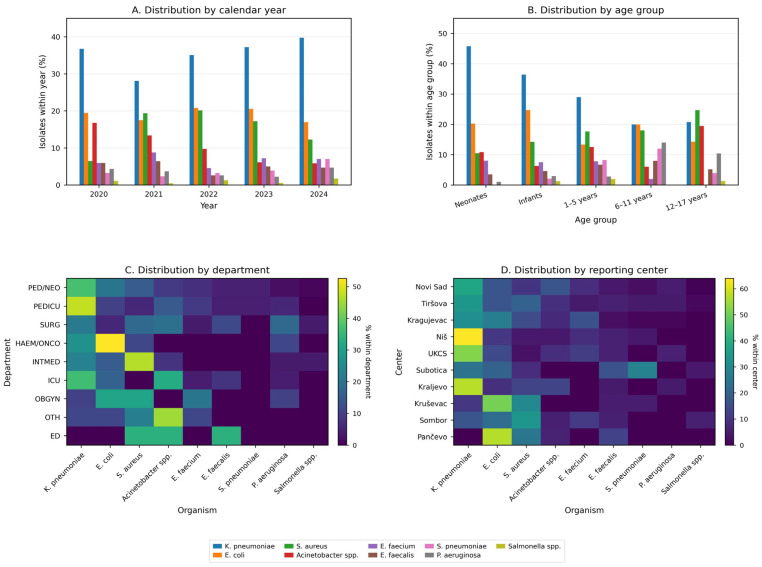
Distribution of bacterial organisms across study strata. Bar charts show proportional organism distribution by calendar year and pediatric age group. Heatmaps show the proportion of each organism within hospital departments and reporting centers; percentages are calculated within each stratum. Low-frequency centers were grouped for inferential analyses. This figure describes organism distribution and does not incorporate antimicrobial susceptibility data.

Among all isolates, *K. pneumoniae* was the most frequently recovered organism (318/907, 35.1%), followed by *E. coli* (172/907, 19.0%), *S. aureus* (137/907, 15.1%), and *Acinetobacter* spp. (96/907, 10.6%). Less frequently isolated organisms included *Enterococcus faecium* (*E. faecium*) (62/907, 6.8%), *Enterococcus faecalis* (*E. faecalis*) (46/907, 5.1%), *Streptococcus pneumoniae* (*S. pneumoniae*) (35/907, 3.8%), *P. aeruginosa* (32/907, 3.5%), and *Salmonella* spp. (9/907, 1.0%). The distribution of bacterial isolates across calendar years, age groups, hospital departments, and centers is shown in Figure 2.

Organism distribution varied across calendar years (χ^2^ = 53.05, df = 32, *p* = 0.011; Cramér’s V = 0.121), age groups (χ^2^ = 135.00, df = 32, *p* < 0.001; Cramér’s V = 0.193), specimen type (χ^2^ = 20.43, df = 8, *p* = 0.0088; Cramér’s V = 0.150), grouped centers (χ^2^ = 132.80, df = 40, *p* < 0.001; Cramér’s V = 0.171), and departments (χ^2^ = 173.77, df = 64, *p* < 0.001; Cramér’s V = 0.155). The effect sizes indicated small to small-to-moderate heterogeneity rather than large stratum effects. The main epidemiological patterns were persistent *K. pneumoniae* predominance, *K. pneumoniae* overrepresentation among neonates, *S. pneumoniae* concentration among children aged 1–5 years, and *P. aeruginosa* overrepresentation in older children. Detailed post hoc residuals and effect-size summaries are provided in Supplementary Table S8.

### 2.3. ESBL and Carbapenemase Findings

ESBL testing was evaluated for *K. pneumoniae* and *E. coli*, the *Enterobacterales* species included in the present ESBL analysis. Among these organisms, ESBL results were available for 464/490 isolates (94.7%), including 302/318 *K. pneumoniae* isolates and 162/172 *E. coli* isolates. Overall, 238/464 tested isolates (51.3%) were ESBL-producing. Carbapenemase testing was recorded for 75/490 *K. pneumoniae*/*E. coli* isolates (15.3%), including 68 *K. pneumoniae* and 7 *E. coli* isolates. Because carbapenemase testing was performed in a selected subset rather than uniformly across all *Enterobacterales* isolates, carbapenemase positivity should be interpreted among tested isolates only and not as a prevalence estimate for the entire study population. Overall ESBL and carbapenemase marker results by tested denominator are summarized in Table 1.

The national WHONET/CAESAR surveillance extract recorded carbapenemase as a categorical positive/negative marker and did not include harmonized carbapenemase class or gene information such as KPC, NDM, VIM, IMP, or OXA-48-like enzymes across all centers. Therefore, type-specific analyses and type-specific geographic comparisons could not be performed at the national-network level. Center-level carbapenemase-marker positivity among tested *K. pneumoniae*/*E. coli* isolates was concentrated in a small number of centers, especially Novi Sad, which accounted for 50/62 carbapenemase-marker-positive *K. pneumoniae*/*E. coli* isolates in the national dataset. However, tested denominators were highly uneven, and these center-level data should be interpreted as descriptive surveillance indicators rather than population-geographic prevalence estimates. Available carbapenemase-type data outside the harmonized national extract were limited to the Novi Sad pediatric center, which contributed the largest number of isolates in the study. In this local subset, among 118 *K. pneumoniae* isolates, 48 were carbapenemase-producing and seven showed confirmed carbapenem resistance without confirmed carbapenemase production. Among carbapenemase-producing *K. pneumoniae* isolates from Novi Sad, metallo-beta-lactamases/NDM predominated (41/48), followed by OXA-48-like enzymes (4/48) and KPC (3/48). Both carbapenemase-producing *E. coli* isolates from Novi Sad were metallo-beta-lactamase/NDM positive. These local data are summarized in Supplementary Table S10 and should be interpreted as high-volume center-specific contextual information rather than a national carbapenemase-type estimate.

In *K. pneumoniae*, ESBL production varied significantly across years, age groups, centers, and hospital departments, but these comparisons should be interpreted as surveillance patterns because clinical case-mix and center-level testing policies were not available. ESBL production was highest in 2020 (69.7%) and 2021 (65.0%), declined in 2022 (44.2%) and 2023 (44.6%), and then increased modestly in 2024 (51.5%). Across age groups, ESBL production was highest in neonates (67.5%) and lowest in adolescents (28.6%).

In *E. coli*, the proportion of ESBL-producing isolates remained stable across years and did not differ significantly across hospital departments. A modest center-level effect was observed, although several center-specific denominators were small.

Carbapenemase-marker analyses were constrained by the limited number of tested isolates, particularly outside Novi Sad and Tiršova. In *E. coli*, carbapenemase testing was too sparse to support meaningful inferential analysis. Accordingly, carbapenemase results are reported as tested-denominator surveillance indicators, not as organism-wide or center-wide prevalence estimates.

### 2.4. Antimicrobial Susceptibility Patterns

Comprehensive organism-specific antimicrobial susceptibility data are provided in Supplementary Table S2. Overall, the highest resistance burden was observed in *K. pneumoniae* and *Acinetobacter* spp., whereas *S. aureus* and enterococci retained susceptibility to several last-line agents. To support rapid interpretation, Table 2 summarizes the main in vitro resistance patterns and antimicrobial categories with retained activity. This table should not be interpreted as a treatment guideline because the study did not include infection syndrome, severity, source control, antimicrobial treatment, pharmacokinetic exposure, drug availability, or outcomes.

**Figure 3:**
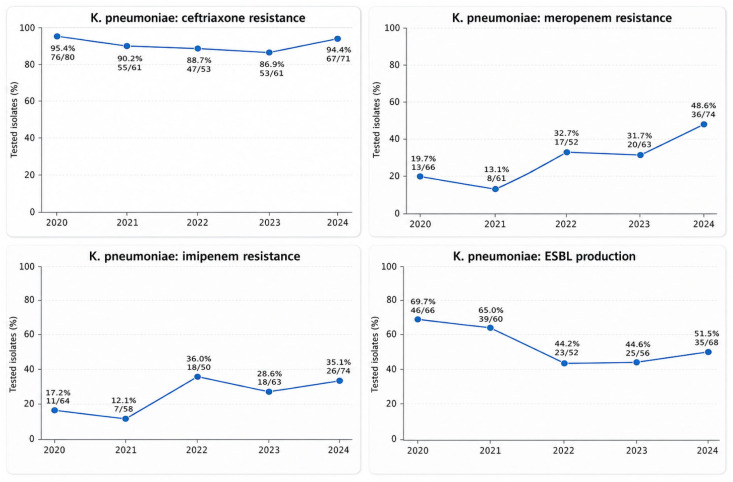
Temporal trends in selected resistance markers among *K. pneumoniae* isolates. Points show the percentage of tested isolates classified as resistant or marker-positive in each calendar year; labels indicate percentage and tested denominator. Ceftriaxone, meropenem, and imipenem panels show EUCAST R-category resistance among isolates with valid AST results. The ESBL panel shows ESBL positivity among isolates with valid ESBL test results. Missing, untested, and not applicable results were excluded from tested denominators.

Among *K. pneumoniae* isolates, ceftriaxone resistance remained consistently very high throughout the study period, ranging from 86.9% to 95.4%, without a significant year effect (*p* = 0.371). In contrast, carbapenem resistance increased over time. Meropenem resistance rose from 19.7% in 2020 to 48.6% in 2024 (χ^2^ = 24.25, df = 4, *p* < 0.001), while imipenem resistance increased from 17.2% to 35.1% over the same period (χ^2^ = 14.50, df = 4, *p* = 0.0059). Ordered-year trend models confirmed significant increasing temporal trends for both meropenem resistance (OR per year 1.48, 95% CI 1.24–1.76, *p* < 0.001) and imipenem resistance (OR per year 1.32, 95% CI 1.10–1.58, *p* = 0.0027). In contrast, ESBL production in *K. pneumoniae* showed a significant decreasing linear trend over time (OR per year 0.80, 95% CI 0.68–0.93, *p* = 0.0049). These temporal patterns are shown in Figure 3.

**Figure 4:**
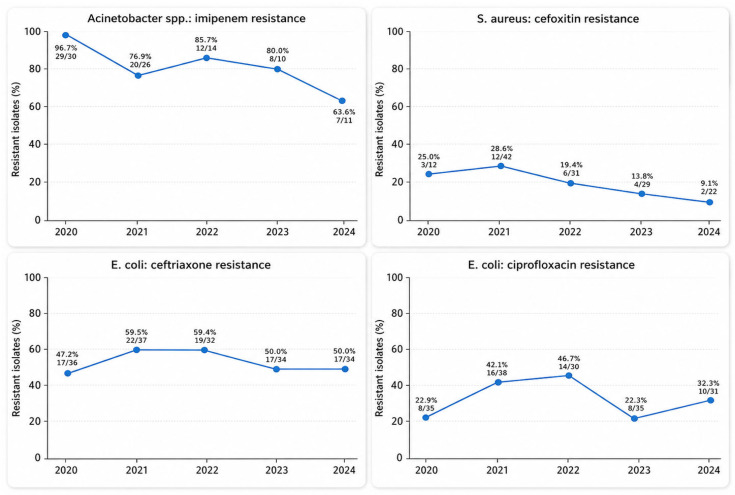
Temporal trends in selected resistance markers among non-*K. pneumoniae* organisms. Points show the percentage of tested isolates with the specified resistance phenotype in each calendar year; labels indicate percentage and tested denominator. Because annual denominators were small for several organism-antimicrobial combinations, these trends should be interpreted descriptively.

Among *E. coli* isolates, resistance to ceftriaxone and ciprofloxacin varied across years, but no significant temporal trends were identified. The proportion of ESBL-producing *E. coli* isolates likewise remained stable over time. Among *Acinetobacter* spp. isolates, carbapenem resistance remained high throughout the study period. Ordered-year trend analysis suggested a decrease in imipenem resistance (OR per year 0.64, 95% CI 0.43–0.96, *p* = 0.029), although the overall year comparison was not statistically significant (*p* = 0.096), and the finding should be interpreted cautiously because annual denominators were small. In *S. aureus*, methicillin resistance inferred from cefoxitin resistance showed a downward pattern over time, but this did not reach conventional statistical significance in the ordered-year trend model (OR per year 0.69, 95% CI 0.48–1.00, *p* = 0.051). Exploratory MDR summaries by organism and year are provided in Supplementary Table S7; because antimicrobial panels differed by organism and year, MDR results were treated as descriptive supporting analyses rather than primary temporal endpoints. These temporal patterns are shown in Figure 4.

### 2.5. Multivariable Analyses

**Figure 5:**
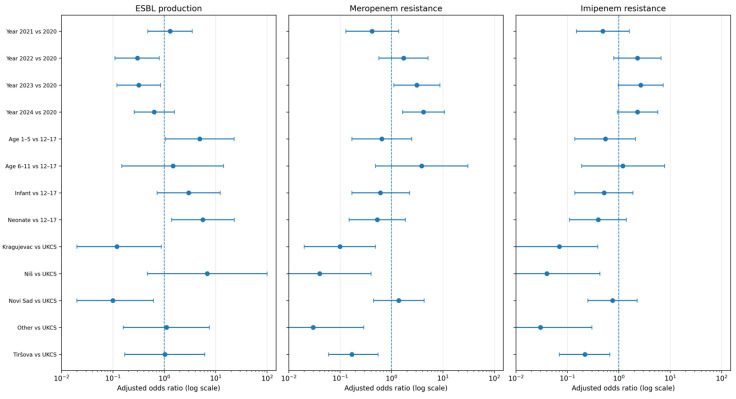
Multivariable logistic regression models for resistance outcomes in *K. pneumoniae*. Points represent adjusted odds ratios and horizontal bars represent 95% confidence intervals on a logarithmic scale. Models were fitted for ESBL production, meropenem resistance, and imipenem resistance among isolates with valid outcome data. Covariates included calendar year, pediatric age group, and grouped center. Reference categories were 2020, age 12–17 years, and UKCS. Detailed estimates are provided in Supplementary Tables S3–S5.

Because *K. pneumoniae* was both the most prevalent organism and the principal contributor to antimicrobial resistance in the dataset, multivariable logistic regression models were constructed for ESBL production, meropenem resistance, and imipenem resistance in this organism. Adjusted associations from multivariable logistic regression models are shown in Figure 5 and detailed in Supplementary Tables S3–S5. A random-intercept mixed-effects model with center as a hierarchical level was considered as a sensitivity approach; however, sparse center strata, highly uneven tested denominators, and separation risk limited stable estimation. Therefore, grouped-center fixed effects were retained as the primary adjusted approach, and center-level results were interpreted descriptively and cautiously.

In the ESBL model, calendar year, age group, and center remained independently associated with the outcome. Relative to the reference year, isolates from 2022 (adjusted OR 0.30, *p* = 0.016) and 2023 (adjusted OR 0.32, *p* = 0.022) had lower odds of ESBL production. Neonatal isolates had substantially higher odds of ESBL production (adjusted OR 5.65, *p* = 0.016), and isolates from children aged 1–5 years also showed increased odds (adjusted OR 4.92, *p* = 0.042). Using UKCS as the reference center, isolates from Novi Sad (adjusted OR 0.10, *p* = 0.014) and Kragujevac (adjusted OR 0.12, *p* = 0.037) had lower adjusted odds of ESBL production. These adjusted associations are shown in Figure 5.

In the meropenem resistance model, later study years remained independently associated with resistance. Compared with the reference year, isolates from 2023 (adjusted OR 3.12, *p* = 0.031) and 2024 (adjusted OR 4.20, *p* = 0.003) had markedly higher odds of meropenem resistance. A similar direction was observed in the imipenem resistance model, although the 2023 and 2024 year terms did not meet conventional statistical significance after adjustment (2023: adjusted OR 2.68, *p* = 0.054; 2024: adjusted OR 2.32, *p* = 0.069). These adjusted associations are shown in Figure 5.

## 3. Discussion

This CAESAR-linked multicenter microbiological surveillance study provides contemporary invasive pediatric isolate data from Serbia. The most robust epidemiological findings were the predominance of *K. pneumoniae*, the concentration of isolates among neonates and infants, and the non-uniform organism distribution across age groups, departments, and centers. The principal AMR signal was the increase in *K. pneumoniae* carbapenem resistance from 2020 to 2024. In contrast, ESBL proportions, carbapenemase positivity, center-level resistance comparisons, and temporal resistance trends should be interpreted as surveillance indicators because testing denominators and laboratory practices were not uniform across all organism-antimicrobial combinations.

The study adds pediatric invasive-isolate evidence from a Western Balkan country to a European AMR landscape in which carbapenem-resistant *K. pneumoniae* is increasingly important. Its value is not that it provides patient-level risk prediction or outcome estimates, but that it identifies a pediatric invasive-isolate signal—especially in neonates and high-risk hospital settings—that can guide future molecular surveillance, local antibiogram development, antimicrobial stewardship priorities, and infection-control review.

The predominance of *K. pneumoniae* is consistent with broader concern regarding Gram-negative pathogens in neonatal and pediatric invasive infections. In the BARNARDS neonatal sepsis study, *Klebsiella*, *E. coli*, and *Enterobacter* were among the main Gram-negative organisms responsible for neonatal sepsis, with widespread antimicrobial resistance and carriage of resistance and virulence determinants [18]. Reviews focused on neonatal sepsis have also emphasized the convergence between neonatal host vulnerability and increasingly resistant or hypervirulent *K. pneumoniae* lineages [19,20]. In the present Serbian dataset, *K. pneumoniae* overrepresentation among neonates therefore fits a clinically plausible pattern rather than representing an isolated local anomaly. It also aligns with Serbian neonatal colonization studies showing substantial carriage of multidrug-resistant Gram-negative organisms and ESBL-producing *Enterobacterales* among hospitalized preterm neonates [14,15].

The divergence between relatively persistent *K. pneumoniae* predominance, declining ESBL production, and increasing carbapenem resistance may reflect a shift in local molecular epidemiology, such as replacement of ESBL-producing lineages by carbapenemase-producing lineages, acquisition of carbapenemase plasmids, or expansion of hospital-adapted high-risk clones. *K. pneumoniae* is recognized as a major reservoir and trafficker of antimicrobial-resistance genes, including carbapenemase genes, and plasmid-mediated dissemination is central to the emergence and spread of carbapenem-resistant *K. pneumoniae* [21,22]. This explanation remains hypothetical in the present study because carbapenemase type, resistance-gene data, plasmid analysis, and strain typing were unavailable.

The locally available Novi Sad carbapenemase-type data support this interpretation as a plausible center-specific hypothesis: among typed carbapenemase-producing *K. pneumoniae* isolates from this high-volume pediatric center, metallo-beta-lactamases/NDM predominated, with smaller OXA-48-like and KPC contributions; both typed carbapenemase-producing *E. coli* isolates from the same center were metallo-beta-lactamase/NDM positive. Because comparable type data were not available for the other participating centers in the national WHONET/CAESAR extract, these data contextualize the main Novi Sad signal but cannot define national carbapenemase-type epidemiology.

The simultaneous decline in ESBL production and rise in carbapenem resistance should not be interpreted as a simple improvement in beta-lactam resistance. Instead, it may represent a transition from cephalosporin-resistance phenotypes toward carbapenemase-associated resistance, altered testing patterns, local clonal replacement, changing referral/case-mix, or implementation of updated susceptibility-testing algorithms. Without molecular testing and standardized longitudinal AST audit data, the study cannot distinguish these mechanisms.

The rise in *K. pneumoniae* carbapenem resistance is the most concerning temporal signal. Meropenem resistance increased from 19.7% in 2020 to 48.6% in 2024, and imipenem resistance increased from 17.2% to 35.1%. These changes remained evident in ordered-year trend models; for meropenem resistance, they also remained significant in multivariable models adjusted for age group and center, while imipenem year effects were directionally similar but borderline after adjustment. The ECDC 2024 EARS-Net report documented that the estimated incidence of carbapenem-resistant *K. pneumoniae* bloodstream infections in the EU increased by more than 60% compared with the 2019 baseline, while the joint WHO Regional Office for Europe/ECDC summary of 2024 surveillance data demonstrated that the burden remained particularly pronounced in southern, central, and eastern parts of Europe [7,8]. The Western Balkans have been specifically described as a region where carbapenem-resistant *Enterobacterales* surveillance gaps must be addressed to complete the European AMR picture [16].

From a therapeutic perspective, carbapenem-resistant *Enterobacterales* are clinically important because active treatment depends on the resistance mechanism, susceptibility profile, infection site, patient age, local drug availability, and pediatric regulatory constraints. Contemporary guidance discusses agents such as ceftazidime-avibactam, meropenem-vaborbactam, imipenem-relebactam, cefiderocol, aminoglycosides, polymyxins, and selected combination strategies for specific resistance mechanisms [23,24,25]. However, pediatric use, toxicity, access, and reimbursement vary by country and institution. Because the present dataset did not include molecular carbapenemase type, treatment, availability, or outcome data, these findings should be used to prioritize local antibiograms, molecular confirmation, and stewardship review rather than to prescribe a universal empirical regimen. Pediatric CRE bacteremia studies and systematic reviews have also shown that CRE bloodstream infection in children is associated with vulnerable host groups, restricted active-treatment choices, and the need for rigorous surveillance and infection-control strategies [26,27].

The resistance profile of *Acinetobacter* spp. also supports concern about hospital-associated Gram-negative pathogens. In this study, *Acinetobacter* spp. accounted for 10.6% of isolates and showed high carbapenem resistance across the study period. *Acinetobacter baumannii* complex organisms can persist in hospital environments, tolerate desiccation, form biofilms, colonize equipment and surfaces, and spread in intensive-care settings [28]. Carbapenem resistance may involve OXA-type carbapenemases, efflux mechanisms, porin alterations, and clonal dissemination. Because this study did not include species-level *Acinetobacter* identification, environmental sampling, or molecular mechanisms, these results should be interpreted as a hospital-surveillance signal requiring local infection-prevention attention. Pediatric intensive-care studies from other regions have similarly associated multidrug-resistant and carbapenem-resistant *A. baumannii* infections with severe disease and high mortality, although the present isolate-level dataset cannot estimate attributable outcomes [29,30].

The Gram-positive findings are less dominant but remain relevant. *S. aureus* accounted for 15.1% of isolates, with cefoxitin resistance in 19.9% of tested isolates as a phenotypic MRSA proxy and preserved vancomycin and linezolid susceptibility among tested isolates. Pediatric MRSA surveillance from the ISPED network reported a large burden of MRSA isolates in children, including neonates and young children, with preserved susceptibility to vancomycin and linezolid but high resistance to several commonly used alternatives [31]. In the Madrid high-complexity pediatric bloodstream-isolate study, methicillin resistance in *S. aureus* was 11.0% and remained stable across the study period, while resistance concerns were more pronounced among Gram-negative organisms [11]. This broader pattern resembles our dataset, where the principal emerging resistance signal is Gram-negative, particularly *K. pneumoniae* carbapenem resistance.

Age heterogeneity should be interpreted mainly as variation in pathogen distribution and healthcare exposure across pediatric age groups, not as proof that age itself independently causes antimicrobial resistance. Neonates contributed 31.5% of isolates and had higher adjusted odds of *K. pneumoniae* ESBL production than adolescents. This age gradient likely reflects a mixture of biological susceptibility, neonatal-unit ecology, exposure to invasive procedures, prematurity-related vulnerability, colonization pressure, and antibiotic-selection pressure. These findings support age-stratified interpretation of pediatric antibiograms rather than reliance on a single pooled pediatric resistance profile. Pediatric bloodstream-infection studies from Beijing and Iran also demonstrate that pathogen distribution and resistance profiles vary by age, underlying disease, and clinical setting, while long-term data from Malawi illustrate how emerging resistance can progressively undermine empirical regimens [32,33,34].

Center-level heterogeneity should also be interpreted cautiously. It may reflect patient case mix, tertiary referral patterns, NICU/PICU capacity, hematology-oncology services, device exposure, antimicrobial prescribing, infection-prevention practices, colonization pressure, outbreak dynamics, and local laboratory testing algorithms. The WHONET-based surveillance extract did not capture center-level multidrug-resistant organism colonization screening, such as routine CRE/MRSA/VRE carriage screening or rectal, nasal, axillary, or groin screening swabs, and it did not capture isolation-policy data, such as single-room isolation, cohorting, personal protective equipment use, or duration of isolation for patients carrying resistant strains. Therefore, the presence, absence, and intensity of colonization-screening and isolation programs could not be analyzed. Prospective multicenter studies should collect these infection-prevention and control variables explicitly and should combine national surveillance with center- and age-specific antibiograms for high-risk departments.

Rather than indicating absence of national pediatric AMR surveillance, these findings show that the existing CAESAR-linked framework should be strengthened by linkage with clinical, molecular, antimicrobial-consumption, colonization-screening, infection-control, and outcome data. The Serbian antibiotic-consumption context provides a plausible ecological background: correlations between consumption of meropenem, ertapenem, ceftriaxone, and levofloxacin and resistance among invasive *K. pneumoniae* and *E. coli* isolates in Serbia have been reported [12]. This association does not prove causality at the patient level, but it supports the stewardship logic that broad-spectrum antibiotic pressure can shape hospital resistance ecology. International pediatric point-prevalence studies from the ARPEC project further show that antimicrobial use is frequent in hospitalized children and varies substantially by ward type and region, supporting the need to link invasive-isolate surveillance with antimicrobial-use indicators [35,36].

The study has several strengths. It uses a multicenter national-surveillance framework, focuses on invasive blood and cerebrospinal fluid isolates rather than mixed specimen types, spans five consecutive years, includes clinically meaningful age groups, and combines descriptive, temporal, and adjusted analyses. The large number of *K. pneumoniae* isolates allowed organism-specific modeling for ESBL production and carbapenem resistance. The supplementary S/I/R table also provides organism-antimicrobial denominators, which is essential because susceptibility testing was not uniform across all antimicrobial combinations.

The most important limitations arise from the retrospective isolate-level surveillance design. First, the unit of analysis was the isolate rather than a clinically adjudicated infection episode; therefore, the dataset could not evaluate illness severity, source control, treatment adequacy, recurrence, mortality, or attributable outcomes. Second, clinical variables such as prematurity, comorbidity, device exposure, previous hospitalization, antimicrobial exposure, colonization status, illness severity, empirical therapy, definitive therapy, source control, length of stay, and mortality were unavailable. Third, AST panels and confirmatory testing algorithms were not uniform across all organism-antimicrobial combinations and may have varied by center and year. Fourth, carbapenemase testing was performed in a selected subset, so carbapenemase positivity should not be interpreted as prevalence in all *Enterobacterales* isolates; test availability and center-specific testing practice may also have contributed to the uneven tested denominators. Fifth, the harmonized national WHONET/CAESAR extract recorded carbapenemase marker status but not carbapenemase type/gene, and local phenotypic carbapenemase-type data were available only for the Novi Sad pediatric center. These local type data are useful for contextualization but should not be extrapolated to all Serbian pediatric centers. Sixth, raw inhibition-zone diameters, MICs, molecular resistance genes, plasmid data, and strain typing were unavailable, preventing central reinterpretation under a single EUCAST breakpoint version and preventing assessment of clonal relatedness. Seventh, colonization-screening, colonization-pressure, and patient-isolation variables were not captured. These limitations mainly affect resistance percentages, center comparisons, carbapenemase estimates, carbapenemase-type interpretation, and temporal trends; organism identification and broad pathogen-distribution findings are less dependent on AST testing policy.

Future surveillance should integrate microbiology, antimicrobial-consumption, patient-level clinical data, colonization-screening data, infection-control variables, and molecular characterization within a One Health framework. For Serbia and the Western Balkans, this is particularly relevant because resistant Gram-negative organisms circulate across hospitals, communities, food systems, animal reservoirs, and cross-border healthcare networks. Coordinated action between hospitals, reference laboratories, antimicrobial-stewardship teams, infection-control services, and public-health authorities is needed to determine whether the observed carbapenem-resistance signal reflects clonal spread, plasmid-mediated dissemination, selective antimicrobial pressure, or changes in testing practice [37].

## 4. Materials and Methods

### 4.1. Study Design and Surveillance Setting

This study was designed as a retrospective multicenter analysis of routine antimicrobial resistance surveillance data from hospitalized pediatric patients in Serbia, reported according to CAESAR invasive-isolate surveillance principles and structured with reference to STROBE reporting guidance for observational studies [9,38]. Surveillance of antimicrobial resistance in Serbia is coordinated through the National Reference Laboratory for Antimicrobial Resistance and the national laboratory network participating in the Central Asian and European Surveillance of Antimicrobial Resistance framework (CAESAR). Serbia has participated in CAESAR since 2013, and the national AMR surveillance network includes laboratories distributed across the country.

Data on identified bacterial isolates and antimicrobial susceptibility results were entered into the WHONET-based national AMR surveillance database, which is used for standardized microbiological data collection, national AMR surveillance, and submission of Serbian AMR data to the CAESAR network. For the present analysis, the harmonized WHONET/CAESAR export contained organism identification, specimen source, center, department, ESBL marker status, carbapenemase marker status, and categorical antimicrobial susceptibility results, but it did not contain harmonized carbapenemase class/gene type or infection-prevention variables across all centers.

The analytic dataset for the present study included eligible pediatric invasive isolates reported by 18 laboratories within this network during the study period: University Clinical Centre of Serbia (UKCS), Institute of Public Health of Vojvodina, Novi Sad, Institute of Public Health Niš, University Clinical Centre Kragujevac, Public Health Institute Sombor, University Children’s Hospital Tiršova, Clinical Hospital Centre “Dr Dragiša Mišović—Dedinje”, Clinical Hospital Centre Zvezdara, Public Health Institute Čačak, Public Health Institute Kraljevo, Public Health Institute Leskovac, General Hospital Kruševac, General Hospital Užice, General Hospital Subotica, Public Health Institute Požarevac, General Hospital Pančevo, Public Health Institute Kikinda, and Public Health Institute Ćuprija “Pomoravlje”. Participating sites represented a mix of tertiary referral/university centers, specialized pediatric services, general hospitals, and public-health institute laboratories serving defined catchment areas. The Institute of Public Health of Vojvodina performs the processing and testing of samples obtained from the Institute for Child and Youth Health Care of Vojvodina, Novi Sad [9].

The study period extended from 1 January 2020 to 31 December 2024. The analysis focused on bacterial isolates recovered from blood cultures and cerebrospinal fluid specimens obtained from hospitalized pediatric patients. Because the objective was to characterize invasive bacterial isolates of high clinical relevance, non-invasive specimens were not included [9]. Surveillance data were checked for consistency and completeness before analysis; however, laboratory-specific audit reports, detailed external quality-assessment performance, center-level staff-training records, and detailed changes in AST algorithms during the study period were not available in the analytic dataset and were therefore not modeled.

### 4.2. Eligibility Criteria and Isolate Selection

Eligible entries were bacterial isolates recovered from blood or cerebrospinal fluid specimens from hospitalized patients aged less than 18 years. To reduce duplicate reporting and align the dataset with surveillance methodology, only the first isolate per patient, bacterial species, and calendar year was included. If the same patient had more than one isolate of the same species within the same calendar year, only the first eligible isolate was retained. Separate bacterial species from the same patient and isolates recovered in different calendar years were considered distinct entries. Because the analysis followed invasive-isolate surveillance methodology, included entries were blood and cerebrospinal fluid isolates belonging to the CAESAR pathogen set. The dataset did not contain clinical adjudication sufficient to distinguish contamination from true infection in every case. Therefore, the study should be interpreted as invasive-isolate microbiological surveillance rather than a clinically adjudicated bloodstream-infection incidence study.

The surveillance pathogen set included *E. coli*, *K. pneumoniae*, *P. aeruginosa*, *Acinetobacter* spp., *S. aureus*, *E. faecalis*, *E. faecium*, *S. pneumoniae*, and *Salmonella* spp., consistent with the CAESAR invasive-isolate surveillance framework [9].

### 4.3. Variables and Resistance Definitions

Available variables included patient sex, age at isolate recovery, age group, specimen type, specimen date/year, organism, center, department, ESBL result, carbapenemase marker result, and categorical antimicrobial susceptibility results. Variables used in multivariable models were calendar year, age group, and grouped center. Department was analyzed descriptively but was not included in final adjusted models because of sparse categories and overlap with center-level structure. Unavailable variables included prematurity, comorbidity, device exposure, previous hospitalization, prior antimicrobial exposure, colonization status, routine CRE/MRSA/VRE screening practice, colonization pressure, screening-swab source, isolation/cohorting policy, single-room use, personal protective equipment use, duration of isolation, illness severity, empirical therapy, definitive therapy, source control, length of stay, mortality, antimicrobial consumption by center, national carbapenemase type/gene, plasmid data, and strain typing.

ESBL production was analyzed among *K. pneumoniae* and *E. coli* isolates with a valid ESBL test result. Carbapenemase production was analyzed among *K. pneumoniae* and *E. coli* isolates with a valid carbapenemase marker result. Isolates with missing, not tested, or not applicable marker results were excluded from the tested denominator for the corresponding marker and were not assumed to be negative. This distinction was particularly important for carbapenemase testing, which was available for a limited and likely selected subset of isolates. The surveillance extract recorded carbapenemase marker status as positive or negative but did not record carbapenemase class or gene type.

For antimicrobial susceptibility summaries, results were reported as susceptible, susceptible with increased exposure, or resistant, corresponding to the EUCAST S, I, and R categories [39]. Percentages in organism-antimicrobial susceptibility summaries were calculated using the number of isolates with valid S/I/R results for the relevant organism-antimicrobial pair as the denominator. Missing or untested combinations were not imputed. For binary resistance analyses, isolates categorized as R were coded as resistant, whereas isolates categorized as S or I were coded as non-resistant. According to EUCAST definitions, category I (susceptible, increased exposure) was retained separately in descriptive tables.

Multidrug resistance (MDR), when reported, was defined according to the international interim proposal as non-susceptibility to at least one agent in three or more antimicrobial categories, adapted to the available organism-specific panels [40]. Because tested antimicrobial panels differed by organism and year, MDR analyses were treated as exploratory and descriptive.

### 4.4. Microbiological Methods and Antimicrobial Susceptibility Testing

Organism identification and antimicrobial susceptibility testing were performed by participating laboratories as part of routine clinical microbiology and national AMR surveillance. Susceptibility testing was conducted using the Kirby–Bauer disk-diffusion method or automated systems, including the VITEK 2 and BD Phoenix platforms, according to local laboratory practice and national surveillance requirements. Where required for confirmation or interpretation, minimum inhibitory concentrations (MICs) were determined using gradient tests or broth microdilution. ESBL and carbapenemase marker results were analyzed as submitted categorical marker results from the national surveillance dataset. Confirmatory testing was performed according to local laboratory and reference-laboratory protocols in use during the study period.

For the Novi Sad pediatric center, where local carbapenemase-type data were available outside the harmonized national extract, carbapenemase detection was performed using phenotypic methods, including disk-diffusion testing with diagnostic tablets (Confirm Kit (KPC, MBL and OXA-48), Rosco Diagnostica NEO-SENSITABS) and rapid immunochromatographic tests (CORIS BioConcept). The disk-diffusion inhibitor approach was used until late 2023 and identified MBL, OXA-48-like, KPC, and other carbapenem-resistance mechanisms. From late 2023 onward, rapid immunochromatographic testing (CORIS BioConcept) was used for carbapenemase detection and enabled detection of KPC, OXA-48, NDM, IMP, and VIM. Because comparable type-level confirmatory-test information was not available in the national extract for all centers, these local type data were analyzed only descriptively.

Antimicrobial susceptibility interpretation followed EUCAST standards as implemented within participating laboratories and recommended by the CAESAR framework [9,39]. The present analysis used submitted categorical S/I/R results rather than reinterpreting raw inhibition-zone diameters or MIC values. Therefore, the results reflect the EUCAST-based routine surveillance categories available in the national dataset. The analytic dataset did not include raw inhibition-zone diameters, MICs for all isolates, detailed confirmatory-test type, molecular gene results, carbapenemase type, or center-specific colonization-screening and isolation-program data; therefore, marker analyses were restricted to valid submitted results and interpreted with denominator caution.

### 4.5. Statistical Analysis

Categorical variables were summarized as counts and percentages. Continuous age was summarized as the median and interquartile range because the pediatric age distribution was right-skewed. Organism distributions were compared across calendar year, age group, specimen type, reporting center, and hospital department using chi-square tests. Fisher’s exact test was used for sparse binary comparisons when appropriate. Where small cell counts limited interpretability, centers or departments were grouped for inferential comparisons. Because multiple exploratory comparisons were performed across surveillance strata, omnibus *p* values and adjusted residuals were interpreted descriptively rather than as causal evidence.

Post hoc adjusted residual analysis was used to identify organism-stratum combinations that contributed most strongly to significant chi-square results. Cramér’s V was reported for chi-square analyses to contextualize practical effect size. Post hoc residual findings were not used alone to support causal or clinical conclusions.

Temporal trends in selected resistance outcomes were evaluated using logistic regression models with calendar year treated as an ordinal predictor. Because *K. pneumoniae* was the most frequent organism and the principal contributor to the observed antimicrobial resistance burden, multivariable logistic regression models were constructed for three binary outcomes in this organism: ESBL production among ESBL-tested isolates, meropenem resistance among isolates with valid meropenem results, and imipenem resistance among isolates with valid imipenem results. Each model included calendar year, age group, and grouped center as covariates, selected a priori because these variables were consistently available and epidemiologically relevant. The reference categories were 2020 for calendar year, adolescents aged 12–17 years for age group, and the University Clinical Centre of Serbia/UKCS group for center. Department was evaluated descriptively but was not included in final multivariable models because of sparse categories and overlap with center-level structure. Model diagnostics included assessment of convergence, sparse cells or separation risk, and collinearity among covariates. Mixed-effects modeling with center as a random effect was considered as a sensitivity approach but was not retained as the primary model because sparse center strata and uneven tested denominators produced unstable estimates.

Adjusted odds ratios, 95% confidence intervals, and two-sided *p*-values were reported. Statistical significance was defined as *p* < 0.05. Analyses and figure generation were performed using Python 3.13.5 within the Visual Studio Code (version 1.123) environment with NumPy, SciPy, statsmodels, and Matplotlib (version 3.11.0).

### 4.6. Ethical and Data-Protection Considerations

The analysis used routinely collected AMR surveillance data without direct patient identifiers. Individual-level informed consent was not sought because the study was retrospective, surveillance-based, non-interventional, and based on anonymized isolate-level records. Data were stored on password-protected institutional systems with access restricted to authorized study personnel. Analyses were performed on anonymized isolate-level records in accordance with applicable national data-protection requirements and institutional policies.

## 5. Conclusions

In this CAESAR-linked multicenter microbiological surveillance study of invasive pediatric isolates from Serbia, *K. pneumoniae* was the predominant pathogen and neonates/infants accounted for most isolates. The clearest AMR signal was the rise in *K. pneumoniae* carbapenem resistance from 2020 to 2024, occurring against a background of frequent ESBL production and marked center-level heterogeneity. Center-level carbapenemase-marker positivity was concentrated mainly in Novi Sad, but testing denominators were uneven; available local carbapenemase-type data from this high-volume pediatric center suggested predominance of metallo-beta-lactamases/NDM among typed carbapenemase-producing *K. pneumoniae* isolates, while national carbapenemase-type inference remains limited because comparable type data were not captured for all centers. Because the dataset consisted of isolate-level surveillance records with non-uniform AST panels, selective carbapenemase testing, limited national type-level data, and no harmonized colonization-screening or isolation-program variables, resistance estimates should be interpreted as surveillance indicators rather than clinical prevalence or outcome estimates. The study’s main contribution is to provide contemporary pediatric invasive-isolate evidence from Serbia and the Western Balkans, supporting center- and age-specific antibiograms, integration of molecular surveillance, linkage with antimicrobial-use, colonization-screening, infection-control, and clinical-outcome data, and coordinated stewardship and infection-control interventions. The observed carbapenem-resistance signal in *K. pneumoniae* should be viewed as a warning sign aligned with wider European AMR trends and as a justification for strengthened pediatric surveillance and public-health action.

## Figures and Tables

**Table 1:** ESBL and carbapenemase marker results by organism and tested denominator.

Organism Denominator	Total Organism Isolates, n	ESBL Tested/Total, n (%)	ESBL-Positive/Tested, n (%)	Carbapenemase Tested/Total, n (%)	Carbapenemase-Positive/Tested, n (%)
*K. pneumoniae* + *E. coli*	490	464/490 (94.7)	238/464 (51.3)	75/490 (15.3)	62/75 (82.7)
*K. pneumoniae*	318	302/318 (95.0)	168/302 (55.6)	68/318 (21.4)	58/68 (85.3)
*E. coli*	172	162/172 (94.2)	70/162 (43.2)	7/172 (4.1)	4/7 (57.1)

**Table 2:** Clinically oriented summary of organism-specific in vitro susceptibility patterns. Percentages are calculated among isolates with valid organism-antimicrobial results. According to EUCAST definitions, category I was interpreted as susceptible, increased exposure.

Organism	Main Resistance Signal	Antimicrobials with Comparatively Retained In Vitro Activity	Interpretive Caution
*K. pneumoniae*	Highest resistance: ceftriaxone 284/311 R (91.3%), gentamicin 267/315 R (84.8%), amoxicillin/clavulanate 248/305 R (81.3%).	Relatively retained in vitro activity: colistin 176/205 S (85.9%); imipenem 198/309 S plus 31/309 I; meropenem 204/316 S plus 18/316 I.	Carbapenem resistance was substantial and increased over time; treatment implications require local guidance and mechanism data.
*E. coli*	Ampicillin 140/167 R (83.8%), ceftriaxone 90/169 R (53.3%), gentamicin 75/172 R (43.6%).	Meropenem 157/170 S (92.4%) plus 3/170 I; imipenem 147/163 S (90.2%) plus 6/163 I; ertapenem 131/145 S (90.3%); amikacin 131/167 S (78.4%).	ESBL production remained frequent, but carbapenem resistance was lower than in *K. pneumoniae*.
*Acinetobacter* spp.	Carbapenem resistance high: imipenem 76/91 R (83.5%), meropenem 76/95 R (80.0%); gentamicin 80/94 R (85.1%).	Colistin 85/86 S (98.8%) showed the highest in vitro activity among tested agents.	Species-level identification and molecular mechanisms were unavailable.
*S. aureus*	Cefoxitin resistance 27/136 R (19.9%) as MRSA proxy.	Vancomycin 137/137 S (100.0%) and linezolid 135/135 S (100.0%).	Resistance burden was lower than that observed among Gram-negative organisms.
*E. faecium*	Ampicillin 61/62 R (98.4%); vancomycin 19/61 R (31.1%).	Linezolid 62/62 S (100.0%); vancomycin 42/61 S (68.9%).	VRE signal requires continued surveillance.
*E. faecalis*	High-level gentamicin 15/45 R (33.3%).	Ampicillin 43/45 S (95.6%), vancomycin 45/46 S (97.8%), linezolid 45/45 S (100.0%).	More favorable profile than *E. faecium*.
*S. pneumoniae*	Erythromycin 13/30 R (43.3%); penicillin 1/30 R (3.3%) with 14/30 I.	Ceftriaxone 27/32 S (84.4%) plus 5/32 I; cefotaxime 14/16 S (87.5%) plus 2/16 I.	Interpretation depends on infection site and EUCAST exposure category.
*P. aeruginosa*	Meropenem 14/32 R (43.8%); ciprofloxacin 12/28 R (42.9%); ceftazidime 12/32 R (37.5%).	Colistin 16/16 S (100.0%); amikacin 27/32 S (84.4%); several antipseudomonal agents remained active with increased exposure (EUCAST category I)	EUCAST category I (susceptible, increased exposure) should be interpreted accordingly
*Salmonella* spp.	Ciprofloxacin 1/7 R (14.3%).	Ceftriaxone 8/8 S (100.0%); meropenem 8/8 S (100.0%).	Small denominator; descriptive only.

## Data Availability

Data availability is restricted due to institutional policies.

## Supplementary Materials

The following supporting information can be downloaded at https://www.mdpi.com/article/10.3390/antibiotics15070694/s1: Table S1: Baseline characteristics of isolate; Table S2: Organism-specific antimicrobial susceptibility profiles; Table S3. Multivariable logistic regression for *K. pneumoniae* ESBL production; Table S4. Multivariable logistic regression for *K. pneumoniae* meropenem resistance; Table S5. Multivariable logistic regression for *K. pneumoniae* imipenem resistance; Table S6: Variable completeness and testing denominators; Table S7: Exploratory multidrug-resistance frequency by organism and year; Table S8: Heterogeneity analyses with effect sizes; Table S9: Carbapenemase marker positivity by grouped center among *K. pneumoniae*/*E. coli* isolates; Table S10: Available local carbapenemase-type data from the Novi Sad pediatric center.
