# Supplementary material for: Antibiotic Resistance Profiles of Invasive Bacterial Isolates from Hospitalized Pediatric Patients in Serbia: A Multicenter Surveillance Study, 2020–2024
Source: Antibiotics (Basel). 2026 Jul 16;15(7):694. doi: 10.3390/antibiotics15070694 (PMC13403949; doi:10.3390/antibiotics15070694)
Supplement: Supplementary file 1 [file antibiotics-15-00694-s001.zip › antibiotics-4423269-supplementary.pdf]

Supplementary Materials

Antibiotic Resistance Profiles of Invasive Bacterial Isolates from Hospitalized Pediatric Patients in Serbia: A Multicenter Surveillance Study, 2020-2024

*Percentages in antimicrobial susceptibility tables are calculated among isolates with valid results for the specified organism-antimicrobial pair. The EUCAST I category is shown separately. Missing, not tested, and not applicable results were excluded from tested denominators.

Supplementary Table S1. Baseline characteristics of isolate

Characteristic	n (%)
Total isolate	907
Sex	
Male	503 (55.5)
Female	404 (44.5)
Specimen type	
Blood	879 (96.9)
Cerebrospinal fluid	28 (3.1)
Age	
Median age, years (IQR)	0.33 (0.03–1.00)
Age group	
Neonate (0–28 days)	286 (31.5)
Infant (29 days to <1 year)	239 (26.4)
1–5 years	255 (28.1)
6–11 years	50 (5.5)
12–17 years	77 (8.5)
Year	
2020	183 (20.2)
2021	217 (23.9)
2022	154 (17.0)
2023	169 (18.6)
2024	184 (20.3)
Center	
Institute of Public Health of Vojvodina, Novi Sad (Center Novi Sad)	316 (34.8)
University Children's Hospital Tiršova (Center Tiršova)	279 (30.8)
University Clinical Centre Kragujevac (Center Kragujevac)	83 (9.2)
Institute of Public Health Niš (Center Niš)	49 (5.4)
University Clinical Centre of Serbia (UKCS)	35 (3.9)
General Hospital Subotica (Center Subotica)	25 (2.8)
Public Health Institute Kraljevo (Center Kraljevo)	23 (2.5)
General Hospital Kruševac (Center Kruševac)	20 (2.2)
Public Health Institute Sombor (Center Sombor)	18 (2.0)
General Hospital Pančevo (Center Pančevo)	16 (1.8)
Other centers*	43 (4.7)
Department	
Pediatrics/Neonatology (PED/NEO)	685 (75.5)
Surgery (SURG)	52 (5.7)
Intensive Care Unit (ICU)	25 (2.8)
Obstetrics/Gynaecology (OBGYN)	10 (1.1)
Pediatric Intensive Care Unit (PEDICU)	70 (7.7)
Haematology/Oncology (HAEM/ONCO)	27 (3.0)
Internal Medicine (INTMED)	26 (2.9)
Emergency Department (ED)	3 (0.3)
Other (OTH)	9 (1.0)

Other centers* Note: Clinical Hospital Centre "Dr Dragiša Mišović – Dedinje", Clinical Hospital Centre Zvezdara, General Hospital Užice, Public Health Institute Čačak, Public Health Institute Leskovac, Public Health Institute Požarevac, Public Health Institute Kikinda, Public Health Institute Ćuprija "Pomoravlje".

Supplementary Table S2. Organism-specific antimicrobial susceptibility profiles

Rows are grouped by organism. S, susceptible; I, susceptible, increased exposure; R, resistant. Percentages are calculated over tested n for each organism-antimicrobial pair.

S2 - K. pneumoniae

Antimicrobial	S n (%)	I n (%)	R n (%)	Tested n
Amikacin	106 (33.3)	0 (0.0)	212 (66.7)	318
Amoxicillin/clavulanate	57 (18.7)	0 (0.0)	248 (81.3)	305
Cefotaxime	13 (11.4)	0 (0.0)	101 (88.6)	114
Ceftazidime	37 (12.2)	24 (7.9)	242 (79.9)	303
Ceftriaxone	21 (6.8)	6 (1.9)	284 (91.3)	311
Ciprofloxacin	155 (50.2)	1 (0.3)	153 (49.5)	309
Colistin	176 (85.9)	0 (0.0)	29 (14.1)	205
Ertapenem	159 (60.0)	0 (0.0)	106 (40.0)	265
Gentamicin	48 (15.2)	0 (0.0)	267 (84.8)	315
Imipenem	198 (64.1)	31 (10.0)	80 (25.9)	309
Levofloxacin	84 (39.3)	19 (8.9)	111 (51.9)	214
Meropenem	204 (64.6)	18 (5.7)	94 (29.7)	316
Piperacillin/tazobactam	93 (32.1)	0 (0.0)	197 (67.9)	290
Tobramycin	6 (3.9)	0 (0.0)	146 (96.1)	152

S2 - E. coli

Antimicrobial	S n (%)	I n (%)	R n (%)	Tested n
Amikacin	131 (78.4)	0 (0.0)	36 (21.6)	167
Amoxicillin/clavulanate	86 (55.5)	0 (0.0)	69 (44.5)	155
Ampicillin	27 (16.2)	0 (0.0)	140 (83.8)	167
Cefotaxime	27 (45.8)	0 (0.0)	32 (54.2)	59
Ceftazidime	84 (52.8)	9 (5.7)	66 (41.5)	159
Ceftriaxone	67 (39.6)	12 (7.1)	90 (53.3)	169
Ciprofloxacin	111 (65.7)	2 (1.2)	56 (33.1)	169
Colistin	66 (100.0)	0 (0.0)	0 (0.0)	66
Ertapenem	131 (90.3)	0 (0.0)	14 (9.7)	145
Gentamicin	97 (56.4)	0 (0.0)	75 (43.6)	172
Imipenem	147 (90.2)	6 (3.7)	10 (6.1)	163
Levofloxacin	59 (56.2)	5 (4.8)	41 (39.0)	105
Meropenem	157 (92.4)	3 (1.8)	10 (5.9)	170
Piperacillin/tazobactam	118 (73.8)	0 (0.0)	42 (26.2)	160
Tobramycin	22 (33.8)	0 (0.0)	43 (66.2)	65

S2 - S. aureus

Antimicrobial	S n (%)	I n (%)	R n (%)	Tested n
Cefoxitin	109 (80.1)	0 (0.0)	27 (19.9)	136
Ciprofloxacin	0 (0.0)	129 (94.2)	8 (5.8)	137
Levofloxacin	0 (0.0)	123 (93.9)	8 (6.1)	131
Linezolid	135 (100.0)	0 (0.0)	0 (0.0)	135
Rifampicin	116 (95.1)	0 (0.0)	6 (4.9)	122
Vancomycin	137 (100.0)	0 (0.0)	0 (0.0)	137

S2 - Acinetobacter spp.

Antimicrobial	S n (%)	I n (%)	R n (%)	Tested n
Amikacin	18 (18.9)	0 (0.0)	77 (81.1)	95
Ciprofloxacin	0 (0.0)	18 (18.9)	77 (81.1)	95
Colistin	85 (98.8)	0 (0.0)	1 (1.2)	86
Gentamicin	14 (14.9)	0 (0.0)	80 (85.1)	94
Imipenem	15 (16.5)	0 (0.0)	76 (83.5)	91
Levofloxacin	13 (16.7)	0 (0.0)	65 (83.3)	78
Meropenem	18 (18.9)	1 (1.1)	76 (80.0)	95
Tobramycin	16 (22.9)	0 (0.0)	54 (77.1)	70

S2 - E. faecium

Antimicrobial	S n (%)	I n (%)	R n (%)	Tested n
Ampicillin	1 (1.6)	0 (0.0)	61 (98.4)	62
High-level gentamicin	9 (14.8)	0 (0.0)	52 (85.2)	61
Linezolid	62 (100.0)	0 (0.0)	0 (0.0)	62
Vancomycin	42 (68.9)	0 (0.0)	19 (31.1)	61

S2 - E. faecalis

Antimicrobial	S n (%)	I n (%)	R n (%)	Tested n
Ampicillin	43 (95.6)	0 (0.0)	2 (4.4)	45
High-level gentamicin	30 (66.7)	0 (0.0)	15 (33.3)	45
Linezolid	45 (100.0)	0 (0.0)	0 (0.0)	45
Vancomycin	45 (97.8)	0 (0.0)	1 (2.2)	46

S2 - S. pneumoniae

Antimicrobial	S n (%)	I n (%)	R n (%)	Tested n
Penicillin	15 (50.0)	14 (46.7)	1 (3.3)	30
Cefotaxime	14 (87.5)	2 (12.5)	0 (0.0)	16
Ceftriaxone	27 (84.4)	5 (15.6)	0 (0.0)	32
Erythromycin	17 (56.7)	0 (0.0)	13 (43.3)	30
Levofloxacin	0 (0.0)	28 (100.0)	0 (0.0)	28

S2 - P. aeruginosa

Antimicrobial	S n (%)	I n (%)	R n (%)	Tested n
Amikacin	27 (84.4)	0 (0.0)	5 (15.6)	32
Cefepime	0 (0.0)	22 (68.8)	10 (31.2)	32
Ceftazidime	0 (0.0)	20 (62.5)	12 (37.5)	32
Ciprofloxacin	0 (0.0)	16 (57.1)	12 (42.9)	28
Colistin	16 (100.0)	0 (0.0)	0 (0.0)	16
Imipenem	0 (0.0)	18 (62.1)	11 (37.9)	29
Levofloxacin	0 (0.0)	13 (56.5)	10 (43.5)	23
Meropenem	18 (56.2)	0 (0.0)	14 (43.8)	32
Piperacillin/tazobactam	0 (0.0)	21 (65.6)	11 (34.4)	32
Tobramycin	14 (58.3)	0 (0.0)	10 (41.7)	24

S2 - Salmonella spp.

Antimicrobial	S n (%)	I n (%)	R n (%)	Tested n
Ceftriaxone	8 (100.0)	0 (0.0)	0 (0.0)	8
Ciprofloxacin	6 (85.7)	0 (0.0)	1 (14.3)	7
Meropenem	8 (100.0)	0 (0.0)	0 (0.0)	8

Supplementary Table S3. Multivariable logistic regression for *K. pneumoniae* ESBL production

Predictor	Adjusted OR	95% CI	p value
Year 2021 vs 2020	1.30	0.48-3.51	0.611
Year 2022 vs 2020	0.30	0.11-0.80	0.016
Year 2023 vs 2020	0.32	0.12-0.85	0.022
Year 2024 vs 2020	0.64	0.26-1.58	0.334
Age group 1–5 years vs 12–17 years	4.92	1.06-22.88	0.042
Age group 6–11 years vs 12–17 years	1.49	0.15-14.27	0.731
Age group Infant vs 12–17 years	3.00	0.73-12.30	0.127
Age group Neonate vs 12–17 years	5.65	1.39-23.00	0.016
Center Kragujevac vs UKCS	0.12	0.02-0.88	0.037
Center Niš vs UKCS	6.85	0.47-100.30	0.160
Center Novi Sad vs UKCS	0.10	0.02-0.62	0.014
Center Other centers vs UKCS	1.11	0.16-7.58	0.917
Center Tiršova vs UKCS	1.03	0.17-6.15	0.974

Note: Intercept omitted. Reference categories: year 2020; age group 12-17 years; center UKCS/University Clinical Centre of Serbia. OR, odds ratio; CI, confidence interval.

Supplementary Table S4. Multivariable logistic regression for *K. pneumoniae* meropenem resistance

Predictor	Adjusted OR	95% CI	p value
Year 2021 vs 2020	0.42	0.13-1.39	0.155
Year 2022 vs 2020	1.72	0.57-5.16	0.333
Year 2023 vs 2020	3.12	1.11-8.76	0.031
Year 2024 vs 2020	4.20	1.65-10.73	0.003
Age group 1–5 years vs 12–17 years	0.65	0.17-2.49	0.534
Age group 6–11 years vs 12–17 years	3.88	0.49-30.76	0.200
Age group Infant vs 12–17 years	0.61	0.17-2.26	0.461
Age group Neonate vs 12–17 years	0.53	0.15-1.87	0.322
Center Kragujevac vs UKCS	0.10	0.02-0.49	0.004
Center Niš vs UKCS	0.04	0.00-0.40	0.007
Center Novi Sad vs UKCS	1.39	0.45-4.31	0.565
Center Other centers vs UKCS	0.03	0.00-0.29	0.002
Center Tiršova vs UKCS	0.17	0.06-0.55	0.003

Note: Intercept omitted. Reference categories: year 2020; age group 12-17 years; center UKCS/University Clinical Centre of Serbia. OR, odds ratio; CI, confidence interval.

Supplementary Table S5. Multivariable logistic regression for *K. pneumoniae* imipenem resistance

Predictor	Adjusted OR	95% CI	p value
Year 2021 vs 2020	0.49	0.15-1.61	0.240
Year 2022 vs 2020	2.31	0.80-6.65	0.120
Year 2023 vs 2020	2.68	0.98-7.34	0.054
Year 2024 vs 2020	2.32	0.94-5.75	0.069
Age group 1–5 years vs 12–17 years	0.55	0.14-2.11	0.381
Age group 6–11 years vs 12–17 years	1.20	0.19-7.82	0.845
Age group Infant vs 12–17 years	0.52	0.14-1.89	0.320
Age group Neonate vs 12–17 years	0.40	0.11-1.41	0.154
Center Kragujevac vs UKCS	0.07	0.01-0.39	0.003
Center Niš vs UKCS	0.04	0.00-0.43	0.007
Center Novi Sad vs UKCS	0.76	0.25-2.29	0.627
Center Other centers vs UKCS	0.03	0.00-0.30	0.002
Center Tiršova vs UKCS	0.22	0.07-0.67	0.008

Note: Intercept omitted. Reference categories: year 2020; age group 12-17 years; center UKCS/University Clinical Centre of Serbia. OR, odds ratio; CI, confidence interval.

Supplementary Table S6. Variable completeness and testing denominators

Available denominators refer to the analytic dataset unless a more specific organism denominator is stated.

Variable / marker	Available or tested n/N (%)	Missing / not tested n/N (%)	Analytic note
Sex	907/907 (100.0)	0/907 (0.0)	Used descriptively.
Age / age group	907/907 (100.0)	0/907 (0.0)	Age was categorized into predefined pediatric groups.
Specimen type	907/907 (100.0)	0/907 (0.0)	Blood or cerebrospinal fluid only.

Specimen date / year	907/907 (100.0)	0/907 (0.0)	Calendar year used for temporal analyses.
Organism	907/907 (100.0)	0/907 (0.0)	CAESAR invasive-isolate pathogen set.
Reporting center	907/907 (100.0)	0/907 (0.0)	Grouped for inferential analyses.
Department/ward code	907/907 (100.0)	0/907 (0.0)	Analyzed descriptively.
ESBL marker among *K. pneumoniae*/*E. coli*	464/490 (94.7)	26/490 (5.3)	Analyzed only among valid tested results.
Carbapenemase marker among *K. pneumoniae*/*E. coli*	75/490 (15.3)	415/490 (84.7)	Selective confirmatory subset; not a prevalence denominator.
Carbapenemase type/gene	0/75 (0.0)	75/75 (100.0)	Carbapenemase class/gene such, as KPC, NDM, VIM, IMP or OXA-48-like, was not recorded.
Colonization screening/isolation program variable	0/907 (0.0)	907/907 (100.0)	Center-specific screening and isolation policies were not captured in the surveillance extract.
Local Novi Sad carbapenemase-type data outside the national extract	50/50 carbapenemase-positive local isolates typed descriptively	Not applicable to national denominator	Single-center contextual data from the largest contributing pediatric center; summarized in Supplementary Table S10 and interpreted as a national carbapenemase-type prevalence estimate.

Supplementary Table S7. Exploratory multidrug-resistance frequency by organism and year

MDR was defined descriptively as non-susceptibility to at least one agent in three or more antimicrobial categories, adapted to the available organism-specific panels.-Because antimicrobial panels differed by organism and year, these results are exploratory surveillance indicators.

Organism	Year	Total isolates, n	MDR-evaluable isolates, n	MDR n (%)
K. pneumoniae	2020	66	66	55 (83.3)
K. pneumoniae	2021	61	61	48 (78.7)
K. pneumoniae	2022	54	54	47 (87.0)
K. pneumoniae	2023	63	63	49 (77.8)
K. pneumoniae	2024	74	74	61 (82.4)
E. coli	2020	36	36	18 (50.0)
E. coli	2021	38	38	23 (60.5)
E. coli	2022	32	32	18 (56.2)
E. coli	2023	35	35	15 (42.9)
E. coli	2024	31	31	14 (45.2)
S. aureus	2020	12	12	1 (8.3)
S. aureus	2021	42	42	1 (2.4)
S. aureus	2022	31	31	0 (0.0)
S. aureus	2023	29	29	0 (0.0)
S. aureus	2024	23	23	0 (0.0)
Acinetobacter spp.	2020	31	31	29 (93.5)
Acinetobacter spp.	2021	29	29	20 (69.0)
Acinetobacter spp.	2022	15	15	12 (80.0)
Acinetobacter spp.	2023	10	10	8 (80.0)
Acinetobacter spp.	2024	11	11	7 (63.6)
E. faecium	2020	11	11	4 (36.4)
E. faecium	2021	19	19	8 (42.1)
E. faecium	2022	7	7	2 (28.6)
E. faecium	2023	12	12	2 (16.7)
E. faecium	2024	13	13	3 (23.1)
E. faecalis	2020	11	11	0 (0.0)
E. faecalis	2021	14	14	0 (0.0)
E. faecalis	2022	4	4	0 (0.0)
E. faecalis	2023	9	9	0 (0.0)
E. faecalis	2024	8	8	0 (0.0)
S. pneumoniae	2020	6	5	0 (0.0)
S. pneumoniae	2021	5	5	0 (0.0)
S. pneumoniae	2022	5	5	0 (0.0)
S. pneumoniae	2023	6	6	0 (0.0)
S. pneumoniae	2024	13	13	0 (0.0)
P. aeruginosa	2020	8	8	3 (37.5)
P. aeruginosa	2021	8	8	3 (37.5)
P. aeruginosa	2022	4	4	1 (25.0)
P. aeruginosa	2023	4	4	0 (0.0)
P. aeruginosa	2024	8	8	6 (75.0)
Salmonella spp.	2020	2	2	0 (0.0)

Salmonella spp.	2021	1	1	0 (0.0)
Salmonella spp.	2022	2	0	NA
Salmonella spp.	2023	1	0	NA
Salmonella spp.	2024	3	3	0 (0.0)

Supplementary Table S8. Heterogeneity analyses with effect sizes

Cramér's V was used to contextualize practical effect size for organism-distribution heterogeneity across strata.

Comparison	χ^2	df	p value	Cramér's V	Interpretation
Calendar year	53.05	32	0.0111	0.121	small-to-moderate
Age group	135.00	32	<0.001	0.193	small-to-moderate
Specimen type	20.43	8	0.0088	0.150	small-to-moderate
Grouped center	132.80	40	<0.001	0.171	small-to-moderate
Department	173.77	64	<0.001	0.155	small-to-moderate

Supplementary Table S9. Carbapenemase marker positivity by grouped center among *K. pneumoniae/E. coli* isolates

The national WHONET/CAESAR surveillance extract recorded carbapenemase as a positive/negative marker but did not record carbapenemase class/gene type for all centers. Therefore, this table summarizes the geographic distribution of carbapenemase-marker positivity, rather than ~~not~~ type-specific carbapenemase epidemiology. Available local carbapenemase-type data from the Novi Sad pediatric center are shown separately in Supplementary Table S10.

Grouped center	Total *K. pneumoniae*/*E. coli* isolates, n	Carbapenemase tested / total, n (%)	Carbapenemase positive / tested, n (%)
Novi Sad	170	57/170 (33.5)	50/57 (87.7)
Tiršova	140	9/140 (6.4)	9/9 (100.0)
Kragujevac	48	2/48 (4.2)	2/2 (100.0)
Niš	36	1/36 (2.8)	1/1 (100.0)
UKCS	23	0/23 (0.0)	0/0 (not tested)
Other centers	73	6/73 (8.2)	0/6 (0.0)

Supplementary Table S10. Available local carbapenemase-type data from the Novi Sad pediatric center

These data were available outside the harmonized national WHONET/CAESAR extract for the Novi Sad pediatric center, whose samples were processed by the Institute of Public Health of Vojvodina. They are provided to contextualize carbapenemase-type distribution in the largest contributing center and should not be interpreted as a national carbapenemase-type prevalence estimate.

Organism / local subset	Carbapenemase result/type	n/N (%)	Interpretive note
K. pneumoniae, total Novi Sad local subset (2020–2024)	Carbapenemase-producing	48/118 (40.7%)	Seven additional carbapenem-resistant *K. pneumoniae* isolates had no confirmed carbapenemase production.
K. pneumoniae, carbapenemase-producing subset	Metallo-beta-lactamase/NDM	41/48 (85.4%)	Predominant local carbapenemase type.
K. pneumoniae, carbapenemase-producing subset	OXA-48-like	4/48 (8.3%)	Minor local contribution.
K. pneumoniae, carbapenemase-producing subset	KPC	3/48 (6.3%)	Smallest local contribution.
E. coli, carbapenemase-producing local subset	Metallo-beta-lactamase/NDM	2/2 (100.0%)	Very small local denominator; descriptive only.

Method note: Local carbapenemase detection used phenotypic methods, including disk-diffusion testing with diagnostic tablets (Confirm Kit (KPC, MBL and OXA-48), Rosco Diagnostica NEO-SENSITABS) and rapid immunochromatographic testing (CORIS BioConcept). The disk-diffusion inhibitor approach was used until late 2023. From late 2023 onward, rapid immunochromatographic testing (CORIS BioConcept) was used for carbapenemase detection-The MBL/NDM designation denotes metallo-beta-lactamase phenotypes. NDM was specified only where rapid immunochromatographic testing enabled specific identification.
